# Restoration of Endothelial Function in *Pparα* ^−/−^ Mice by Tempol

**DOI:** 10.1155/2015/728494

**Published:** 2015-11-15

**Authors:** Neerupma Silswal, Nikhil Parelkar, Jon Andresen, Michael J. Wacker

**Affiliations:** University of Missouri-Kansas City School of Medicine, Kansas City, MO 64108, USA

## Abstract

Peroxisome proliferator activated receptor alpha (PPAR*α*) is one of the PPAR isoforms belonging to the nuclear hormone receptor superfamily that regulates genes involved in lipid and lipoprotein metabolism. PPAR*α* is present in the vascular wall and is thought to be involved in protection against vascular disease. To determine if PPAR*α* contributes to endothelial function, conduit and cerebral resistance arteries were studied in *Pparα* ^−/−^ mice using isometric and isobaric tension myography, respectively. Aortic contractions to PGF_2*α*_ and constriction of middle cerebral arteries to phenylephrine were not different between wild type (WT) and *Pparα* ^−/−^; however, relaxation/dilation to acetylcholine (ACh) was impaired. There was no difference in relaxation between WT and *Pparα* ^−/−^ aorta to treatment with a nitric oxide (NO) surrogate indicating impairment in endothelial function. Endothelial NO levels as well as NO synthase expression were reduced in *Pparα* ^−/−^ aortas, while superoxide levels were elevated. Two-week feeding with the reactive oxygen species (ROS) scavenger, tempol, normalized ROS levels and rescued the impaired endothelium-mediated relaxation in *Pparα* ^−/−^ mice. These results suggest that *Pparα* ^−/−^ mice have impaired endothelial function caused by decreased NO bioavailability. Therefore, activation of PPAR*α* receptors may be a therapeutic target for maintaining endothelial function and protection against cardiovascular disease.

## 1. Introduction

Peroxisome proliferator activated receptor alpha (PPAR*α*) is one of the PPAR isoforms belonging to the nuclear hormone receptor superfamily. PPAR*α* binds to specific DNA sequences termed PPAR response elements (PPRE) after coupling with retinoid X receptor (RXR) and functions primarily to alter gene expression. PPAR*α* is mainly expressed in heart, liver, kidney, and muscle where it regulates genes involved in lipid and lipoprotein metabolism. PPAR*α* is also present in endothelial and smooth muscle cells of the vascular wall and is thought to be involved in protection against vascular disease [1, 2].

The endothelium serves as an important regulator of vascular smooth muscle relaxation via release of vasorelaxing factors such as nitric oxide (NO). Endothelial NO production is principally controlled by endothelial NO synthase (eNOS) and the bioavailability of NO can be altered by the presence of reactive oxygen species (ROS) which can react with NO to form peroxynitrite [3]. There have been no direct studies of vascular function in* Pparα* ^−/−^ mice; however, treatment with PPAR*α* agonists improved aortic function in mice [4] and restored eNOS activity in hypertensive rats [5]. These findings suggest that PPAR*α* has a protective role in the cardiovascular system via modulation of vascular function, but the mechanism of action remains unknown.

In this study we investigated the endothelial function of* Pparα* ^−/−^ mice using isobaric and isometric tension myography of the middle cerebral artery (MCA) and the aorta, respectively. Superoxide levels were measured in aortas using lucigenin-enhanced chemiluminescence. Aortic NO was measured by DAF-FM fluorescence and eNOS expression was determined by Western blot. In an attempt to restore endothelial function, mice were fed drinking water supplemented with tempol, a superoxide dismutase mimetic.

## 2. Materials and Methods

### 2.1. Animals and Reagents

Male C57BL/6J (WT) and PPAR*α* deficient (*Pparα* ^−/−^) mice (aged 12–16 weeks) were purchased from The Jackson Laboratory (Bar Harbor, ME). Mice were euthanized by CO_2_ inhalation and decapitated prior to tissue harvesting. Deletion of the* Pparα* gene was confirmed by using primer sequences available from The Jackson Laboratory database using standard PCR conditions. For endothelial function restoration studies, WT and* Pparα* ^−/−^ mice were fed regular drinking water and tempol supplemented water at a dose of 1 mM for 2 weeks. All reagents were sourced from Sigma (St. Louis, MO) unless otherwise noted. The Animal Care and Use Committee at the University of Missouri-Kansas City approved all protocols.

### 2.2. Isometric Tension Myography: Aorta

The thoracic aortas from* Pparα* ^−/−^ mice and age-matched WT mice were rapidly excised and placed in ice-cold Hank's buffered saline solution (HBSS, Invitrogen, Carlsbad, CA) where blood, fat, and excess connective tissues were carefully removed. Segments 3-4 mm in length were mounted on pins in chambers of DMT 610 M wire myograph system (Danish Myo Technology A/S, Aarhus N, Denmark) containing Krebs buffer saturated at 37°C with a gas mixture containing 20% O_2_/5% CO_2_/75% N_2_ (Airgas Mid-South Inc., Tulsa, OK). Arterial rings were progressively stretched to 0.75 g equivalent force passive tension in 0.1 g steps and allowed to equilibrate for 45 minutes described previously [6]. Aortic rings were exposed to isotonic KCl (40 and 80 mM) to assess the quality of the preparation. A concentration response curve to prostaglandin F_2*α*_ (PGF_2*α*_) (10^−9^–10^−4^ M) was determined. To assess the endothelial function, vessels were precontracted with 10^−5^ M PGF_2*α*_, and concentration response curve to acetylcholine (Ach) (10^−9^–10^−4^ M) was performed. Similarly, a concentration response curve to sodium nitroprusside (SNP) (10^−9^–10^−4^ M) was carried out after precontraction with 10^−5^ M PGF_2*α*_ to assess smooth muscle function. Contraction response curves to serotonin (5-HT) (10^−9^–10^−5^ M) were also performed. Vessels were rinsed once with fresh Krebs every 15 min and several times after concentration response curves. Relaxation to ACh was measured in PGF_2*α*_ preconstricted aortas following endothelial denudation, which was performed by gently rubbing the vessel lumen with forceps. Force changes were recorded using an ADinstruments (Colorado Springs, CO) PowerLab 4/30 and associated LabChart Pro software (v6.1) running on a standard Windows XP computer platform.

### 2.3. Isobaric Vessel Studies: MCA

Brains were quickly removed and placed in ice-cold Hank's buffered saline solution (HBSS, Invitrogen, Carlsbad, CA). MCAs were studied in a pressurized artery myograph (DMT-USA, Ann Arbor, MI) as previously described [6–9]. Briefly, MCAs were carefully dissected away from the brain, cleared of blood and pia mater, mounted on glass micropipettes, and pressurized to 70 mmHg with Krebs buffer (in mM: 119 NaCl, 4.7 KCl, 0.24 NaHCO_3_, 1.18 KH_2_PO_4_, 1.19 MgSO_4_, 5.5 glucose, and 1.6 CaCl_2_). Elevated external K^+^ buffers were made isotonic by replacement of NaCl with KCl on an equimolar basis. MCAs were exposed to isotonic KCl (40 and 80 mM) to assess the quality of the preparation. A concentration response curve to phenylephrine (PE) (10^−9^–10^−4^ M) was determined to assess constriction. To evaluate endothelial function, vessels were preconstricted with 10^−5^ M PE, and a concentration response curve to ACh and bradykinin (10^−9^–10^−4^ M) was performed.

### 2.4. DAF-FM Staining

NO levels in aortic rings were assessed and imaged using the membrane-permeable dye 4-amino-5-methylamino-2′,7′-difluorofluorescein diacetate (DAF-FM; Invitrogen, Carlsbad, CA). WT and* Pparα* ^−/−^ aortic rings (~3 mm in length) were cleaned of fat and connective tissue and equilibrated for 30 min in HBSS at room temperature. DAF-FM (10 *μ*M) was then added to the buffer for 30 min in the dark. The aortic rings were washed two times with fresh HBSS buffer and immediately snap-frozen with OCT embedding compound in isopentane prechilled with liquid nitrogen. Frozen rings were then cut into 10 *µ*m sections and imaged by epifluorescence microscopy using an Olympus IX71 (Center Valley, PA) inverted microscope fitted with Hamamatsu ORCA-R2 CCD camera (Bridgewater, NJ), Sutter LB-XL light source (Novato, CA), and Semrock filters (Rochester, NY) with optimized excitation and emission wavelengths (DAF-FM, 495/519 nm). All images were captured under constant exposure time and gain. The fluorescence intensity of the endothelial cell layer was quantified using Slidebook (Intelligent Imaging Innovations Inc., Denver, CO). The endothelial regions from each ring were selected randomly and quantified via mean fluorescence intensity.

### 2.5. Western Blotting

Briefly, flash-frozen aortic segments were homogenized by a glass homogenizer in protein extraction buffer [1% SDS, 10 mM EDTA, and complete mini protease inhibitor cocktail (Roche Diagnostics, Basel, Switzerland)]. Samples were heated to 85°C for 15 min and centrifuged at 15,000 ×g for 15 min at 4°C. Protein concentration of the supernatants was determined by use of the DC Protein Assay (Bio-Rad Laboratories, Hercules, CA). Samples were diluted with 6x Laemmli buffer (30% glycerol, 50 mM EDTA, 0.25% bromphenol blue, and 10%  *β*-mercaptoethanol) and heated to 85°C before loading. The protein samples (40 *µ*g) were run on a 7.5% sodium dodecyl sulphate-polyacrylamide gel electrophoresis (SDS-PAGE) and the separated proteins were transferred to polyvinyl difluoride membranes. Nonspecific-binding sites were blocked with 5% BSA. The membranes were then incubated with the monoclonal antibody to eNOS (1 : 1000) (BD Transduction Laboratories). Afterwards, membranes were incubated with anti-rabbit or anti-mouse immunoglobulin (IgG) peroxidase-conjugated secondary antibodies (1 : 2500). Bound antibodies were detected by ECL Western blotting detection kit (Amersham Life Sciences, Arlington Heights, IL, USA). To ensure equal protein loading, membranes were stripped and reprobed with anti-*α*-actin antibody (1 : 5000; Abcam).

### 2.6. Lucigenin-Enhanced Chemiluminescence

Aortic rings (2 mm in length) from water and tempol supplemented WT and* Pparα* ^−/−^ mice were preincubated for 45 min at 37°C in HBSS. Aortic rings were then transferred into the wells of a 96-well plate, each of which contained 200 *μ*L of HBSS-based assay solution consisting of 5 *μ*M lucigenin. The light reaction between superoxide and lucigenin was detected in a microplate luminometer (GloMax, Promega, USA) and tissue-dependent photon emission per second per well was monitored over a 20 min period as described earlier [10]. At the completion of the assay, aortic rings were dried in a 60°C oven for 24 h, enabling superoxide production to be normalized to dry tissue weight.

### 2.7. Statistics

Data were plotted and expressed as means ± SEM and *n*-values are detailed in the legends of the figures. In myograph experiments, changes in isometric tension are expressed as % relaxation. Changes in the diameter of pressurized MCAs were calculated as % dilation as previously described [11]. Two-factor ANOVA was used to determine differences between concentration response curves. A Bonferroni* post hoc* test was used for multiple comparisons between concentration response curves. A one-factor ANOVA and Tukey's multiple comparison* post hoc* tests were used for analyzing Western blot data. A *t*-test was used to compare values from the superoxide assay as well as DAF-FM analysis. Data were plotted and statistics computed with Graphpad Prism (v5.01, San Diego, CA). Significance was accepted at *p* < 0.05.

## 3. Results

### 3.1. *Pparα* ^−/−^ Mice Aortas: Vascular Function

**Figure 1:**
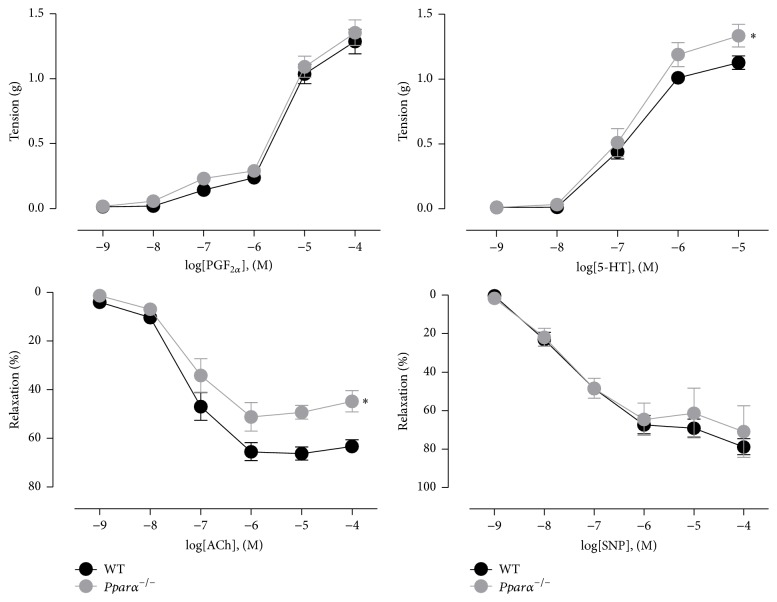
Impaired endothelium-dependent relaxations in aortas of* Pparα* ^−/−^ mice. Contractile response curves to prostaglandin F_2*α*_ (PGF_2*α*_) and 5-hydroxytryptamine (5-HT) and relaxation response curves to acetylcholine (ACh) and sodium nitroprusside (SNP) on PGF_2*α*_-precontracted aortic rings from wild type (WT) and* Pparα* ^−/−^ mice. *∗* denotes statistical significance from WT mice (*p* < 0.05; *n* = 5 animals). Data are means ± SEM.

To investigate the role of PPAR*α* in the vasculature, we studied aortic function in* Pparα* ^−/−^ mice as compared to age-matched WT control mice. We first examined the contractile response to PGF_2*α*_ (10 nM to 100 *µ*M) and there was no difference in aortic ring contraction between* Pparα* ^−/−^ and WT mice (*p* > 0.05; Figure 1). Contraction to serotonin (5-HT) was higher in* Pparα* ^−/−^ aortic rings compared to WT (*p* < 0.05: Figure 1). While PGF_2*α*_ elicits contraction alone, 5-HT can release NO from the endothelium and induce smooth muscle contraction. Thus, the increase in contraction indicated that there was endothelial dysfunction in* Pparα* ^−/−^ mice. As confirmation of this, endothelium-dependent relaxation to ACh was impaired by 29% in* Pparα* ^−/−^ aortic rings (*p* < 0.05; Figure 1). The NO surrogate, SNP, was used to determine if the impaired relaxation was due to endothelial or smooth muscle dysfunction. The relaxation response to SNP was similar in both groups of mice (*p* > 0.05; Figure 1) indicating that* Pparα* ^−/−^ mice aortas have impaired endothelial-dependent relaxations. To confirm that relaxations to ACh were endothelium-dependent in* Pparα* ^−/−^ aortas, ACh responses were assessed in arteries denuded of endothelium. As expected, in endothelium-denuded* Pparα* ^−/−^ aortas ACh did not induce relaxations indicating that as in WT mice ACh stimulates the endothelium to induce smooth muscle relaxation. Together with the SNP responses these data indicate that the impairment was in endothelial function.

### 3.2. *Pparα* ^−/−^ Mice MCA: Vascular Function

**Figure 2:**
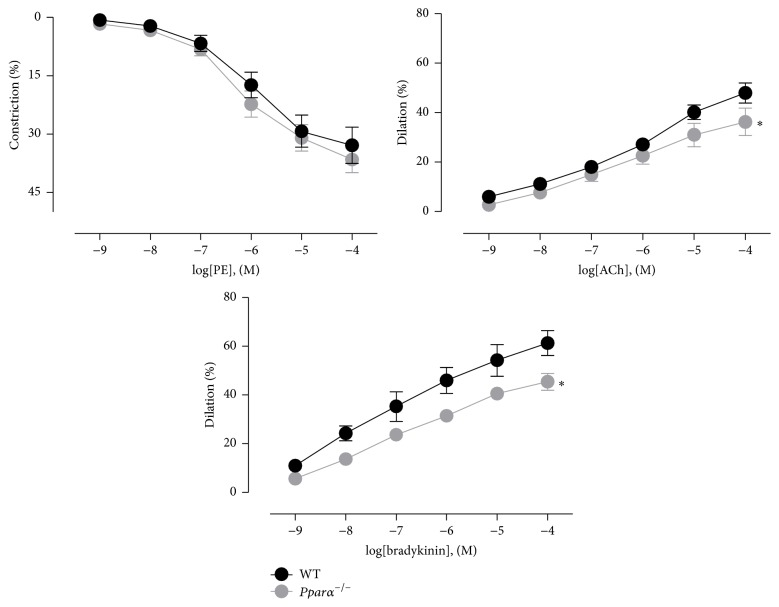
Impaired endothelium-dependent dilations in MCAs of* Pparα* ^−/−^ mice. Contractile response curves to phenylephrine (PE) and dilatory response to ACh and bradykinin on PE- preconstricted middle cerebral artery (MCA) of WT and* Pparα* ^−/−^ mice using isobaric myography. *∗* denotes statistical significance from WT mice (*p* < 0.05; *n* = 5 animals). Data are means ± SEM.

In addition to the aorta, we also examined cerebral resistance artery function using the MCA. Compared to WT mice, the constriction responses to PE were unaltered in* Pparα* ^−/−^ mice (*p* > 0.05; Figure 2), but dilatory responses to ACh and bradykinin were significantly reduced (*p* < 0.05; Figure 2). In the MCA, both ACh and bradykinin can induce dilation by multiple mechanisms including stimulation of eNOS and NO production, PGI2, epoxyeicosatrienoic acid, and release of endothelium-derived hyperpolarizing factor (EDHF). Thus, while reduced NO bioavailability is likely responsible for the impaired dilation observed in* Pparα* ^−/−^ mouse MCAs, it remains possible that other dilatory pathways were affected as well, although EDHF is highly resistant to oxidative stress. In total, these data indicate that endothelial function is impaired in both conduit and cerebral resistance arteries in* Pparα* ^−/−^ mice.

### 3.3. NO Levels in Endothelium

**Figure 3:**
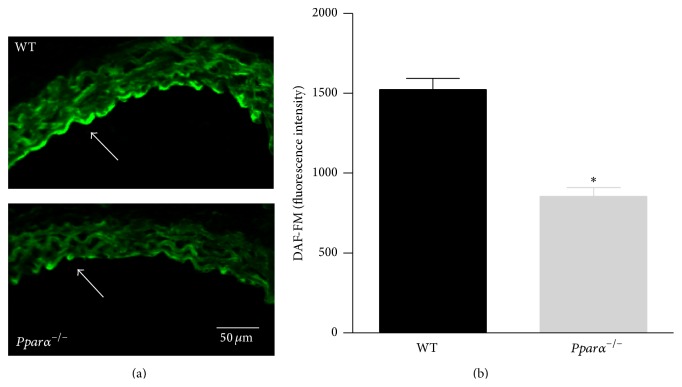
Decreased NO bioavailability in* Pparα* ^−/−^ mice. (a) Aortic rings from WT (top) and* Pparα* ^−/−^ (bottom) were sectioned and stained with DAF-FM to detect NO. The white arrow indicates the endothelial layer where there is increased fluorescence in WT compared to* Pparα* ^−/−^ aorta. (b) Summary data of the DAF-FM fluorescence of the endothelial layer of WT and* Pparα* ^−/−^ aortas. *∗* denotes statistical significance from WT mice (*p* < 0.05; *n* = 4 rings from 2 pairs of mice). Data are means ± SEM.

We imaged and estimated NO levels from the endothelial layer of aortic sections from WT and* Pparα* ^−/−^ mice using DAF-FM fluorescence. We found that the basal level of NO was reduced in aortas of* Pparα* ^−/−^ versus WT mice (*p* < 0.05; Figure 3). The reduction in NO levels is consistent with the findings that stimulated release of NO from endothelium was also impaired in aortas and MCAs of* Pparα* ^−/−^ mice. Together these data affirm that* Pparα* ^−/−^ mouse arteries exhibit reduced NO bioavailability leading to impaired vascular function.

### 3.4. Expression of eNOS

**Figure 4:**
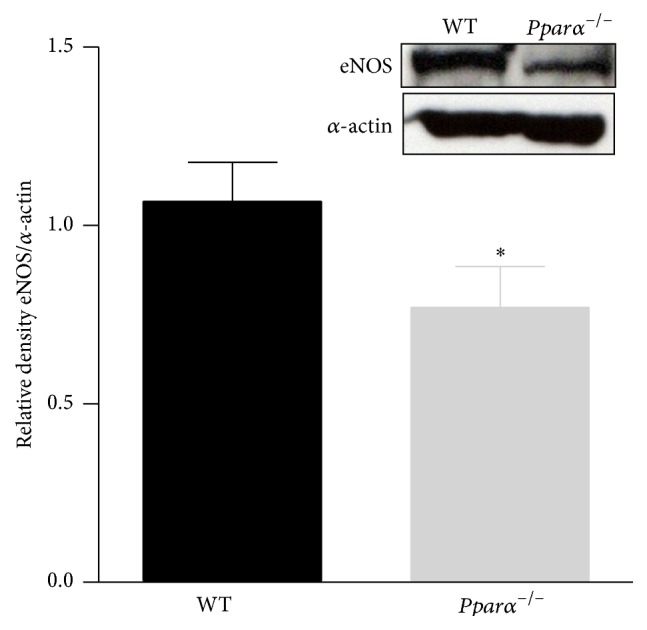
Decreased expression of eNOS in* Pparα* ^−/−^ mice. Western blot data showing the expression level of eNOS in aorta of* Pparα* ^−/−^ and WT mice. *∗* denotes statistical significance from WT mice (*p* < 0.05; *n* = 3 animals). Data are means ± SEM.

Since PPARs regulate gene expression and eNOS produce the endothelial relaxing factor, NO, we compared the expression of total eNOS protein extracted from aortas of* Pparα* ^−/−^ and WT mice. After normalization to *α*-actin, eNOS protein was 28% lower in aortas of* Pparα* ^−/−^ compared to WT mice (*p* < 0.05; Figure 4). Thus, Western blot data suggest that impaired endothelial function observed in* Pparα* ^−/−^ mice is due, in part, to reduced expression of eNOS and impaired NO bioavailability.

### 3.5. Tempol Treatment, Superoxide Levels, and Vascular Function

**Figure 5:**
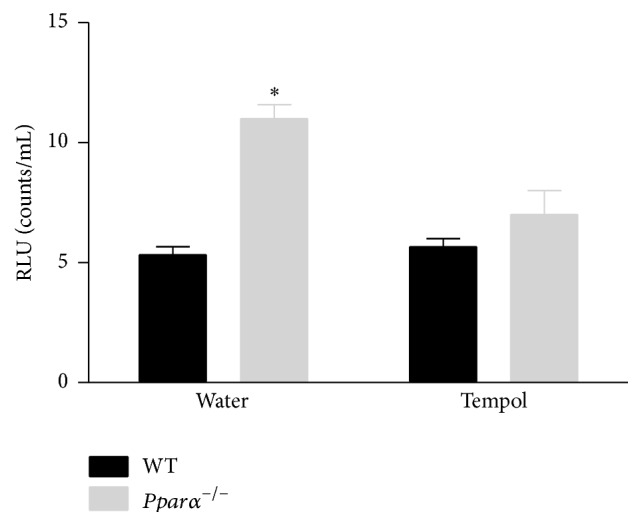
Elevated superoxide levels in* Pparα* ^−/−^ mice are reduced following tempol treatment. Lucigenin- (5 *µ*M) enhanced chemiluminescence in mouse aortic segments from WT and* Pparα* ^−/−^ mice after two weeks of treatment with water and tempol. Elevated levels of superoxide in* Pparα* ^−/−^ mice were reduced to the level of WT mice after tempol treatment. *∗* denotes statistical significance from water treated WT mice (*p* < 0.05; *n* = 3 animals). Data are means ± SEM.

**Figure 6:**
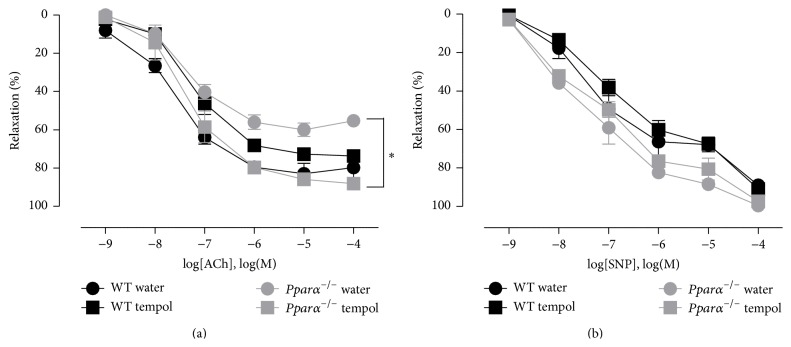
Tempol restored aortic relaxation in* Pparα* ^−/−^ mice. (a) Relaxation response curves to ACh and (b) SNP in WT and* Pparα* ^−/−^ mice after two weeks of water and tempol treatment. *∗* denotes statistical significance between tempol and water treated* Pparα* ^−/−^ mice (*p* < 0.05; *n* = 3 animals). Data are means ± SEM.

To determine if the impaired endothelial function and decreased NO bioavailability were due to scavenging by reactive oxygen species (ROS), we examined superoxide levels in aortas of* Pparα* ^−/−^ and WT mice fed either tempol or water for two weeks. Lucigenin-enhanced chemiluminescence revealed that superoxide levels were twofold greater in* Pparα* ^−/−^ than WT mice (*p* < 0.05; Figure 5). Tempol treatment normalized ROS levels in the aortas of* Pparα* ^−/−^ mice (*p* < 0.05; Figure 5). The decrease in superoxide levels as measured by lucigenin after tempol treatment verified that tempol was effectively working as SOD mimetic. To determine the role of superoxide in impairing endothelium-dependent relaxation, aortic responses to ACh were measured from both the water and tempol treated WT and* Pparα* ^−/−^ mice. Tempol supplementation improved the ACh response in* Pparα* ^−/−^ mice aortas restoring it to WT levels (*p* < 0.05; Figure 6). Similar to our previous findings, there was no difference to SNP mediated relaxations (*p* > 0.05; Figure 6). Since function was rescued by treatment with a ROS scavenger, these results suggest that the impaired endothelial function and reduced NO in aortas of* Pparα* ^−/−^ mice were due to elevated ROS levels. While tempol can scavenge other ROS besides superoxide, superoxide directly reacts with NO to form peroxynitrite thereby reducing NO bioavailability and therefore is the most likely ROS involved in the dysfunction, although other species cannot be ruled out to also play a role in the endothelial impairment.

## 4. Discussion

PPARs are critical for the metabolism of lipids and lipoproteins. These receptors are also known to play a critical role in the regulation of normal vascular function, but the mechanisms are largely unknown. Mice with endothelial cell-specific dominant negative mutations of* Pparγ* demonstrated endothelial dysfunction in response to high fat diet while mice with smooth muscle cell-specific dominant negative mutations in* Pparγ* have shown compromised NO-mediated vasodilation as well as systolic hypertension [12, 13]. Similarly, vascular endothelial cell-specific* Pparδ* ^−/−^ mice also displayed significantly impaired endothelium-dependent and endothelium-independent relaxations in aorta and carotid arteries [14]. However, the effects of* Pparα* ^−/−^ deficiency on mouse vascular bed are unknown.

This is the first study to directly examine vascular function in* Pparα* ^−/−^ mice. Specifically, we explored the role of PPAR*α* in vascular smooth muscle and endothelial function as well as the relationship to ROS in the vasculature. We found that vascular smooth muscle contractile function was normal in* Pparα* ^−/−^ mice whereas endothelial function was impaired in both conduit and cerebral resistance arteries. Expression of eNOS was reduced in conduit arteries of* Pparα* ^−/−^ mice, superoxide levels were elevated, and NO levels were decreased. Furthermore, the ROS scavenger, tempol, normalized superoxide levels and restored endothelium-dependent relaxation in aortas indicating that decreased NO bioavailability caused by ROS resulted in the endothelial dysfunction.

Impairment of endothelial function can occur due to many reasons; however, a prominent cause is increased ROS, particularly superoxide, reducing the bioavailability of NO released by the endothelium. This mechanism is particularly important in the endothelial impairment brought on by diseases such as diabetes or atherosclerosis [15–18]. It is possible that activation of PPAR*α* is necessary to maintain a steady state level of ROS which explains why superoxide was elevated and NO reduced in the* Pparα* ^−/−^ aorta. Treatment with the PPAR*α* agonist, fenofibrate, has been shown to improve endothelial-mediated aortic relaxation in mice [4]. The impairment of NO bioavailability we observed in* Pparα* ^−/−^ arteries supports these findings and may help to provide a mechanism for the improvement that was observed in this previous study. Similar to our findings, treatment with a superoxide scavenger restored endothelial function in the basilar artery of endothelial cell-specific* Pparγ* ^−/−^ mice fed a high fat diet [12]. In addition, endothelial dysfunction in aortas of endothelial cell-specific* Pparδ* ^−/−^ mice was also attributed to elevated ROS levels [14]. Thus, there is mounting evidence that all three PPAR receptors are important for vascular protection. Elucidating particular roles for each receptor in individual vascular beds and in various disease settings is worthy of future study.

The endothelial dysfunction that we observed in* Pparα* ^−/−^ mice could also be due, in part, to the decrease in eNOS expression. It is possible that PPAR*α* plays a direct role in altering eNOS levels since agonists of PPAR*α* increased eNOS expression in bovine aortic endothelial cells [19]. Alternatively, the increases in superoxide that we observed may be responsible for reduced eNOS expression or activity. For example, treatment with activators of PPAR*α* improved eNOS activity by decreasing ROS in a hypertensive rat model [5]. In aged mice with reduced endothelial function, treatment with tempol restored endothelium-dependent dilation by increasing eNOS and thus NO bioavailability [20]. Similarly, in mesenteric arteries of aging rats, increased peroxynitrate levels were responsible for decreased endothelium-dependent relaxations which were normalized after tempol treatment [21]. It is also possible that through substrate deletion the inducible form of nitric oxide synthase (iNOS) could also have contributed to the endothelial dysfunction that we observed. Nevertheless, we did not observe any impairment in agonist-induced smooth muscle contraction, which occurs when iNOS is active [22–24]. Thus, while it is possible that iNOS is activated in arteries of* Pparα* ^−/−^ mice it did not appear to contribute to the impaired endothelial function we observed.

The effects we observed in* Pparα* ^−/−^ mice have potentially significant consequences for cardiovascular disease. In this study, we found that endothelial function was selectively impaired in both conduit and cerebral resistance arteries of* Pparα* ^−/−^. Impaired relaxation of a conduit artery such as the aorta may lead to increases in afterload on the left ventricle and ultimately to cardiac hypertrophy and remodeling. For example, aortic constriction in* Pparα* ^−/−^ mice resulted in increased cardiac hypertrophy compared to WT mice [25] and* Pparα* ^−/−^ mice on a high salt diet had an increased heart weight/body weight ratio compared to WT mice [26] potentially demonstrating a greater afterload effect in* Pparα* ^−/−^.

General impairment of resistance arteries may also lead to increases in systemic blood pressure as well as decreases in blood flow to specific organs. For example, angiotensin-infused* Pparα* ^−/−^ mice displayed elevated mean arterial pressure compared to WT, and activation of PPAR*α* attenuated Ang II-induced hypertension in WT mice [27]. Interestingly, Obih and colleagues did not observe a basal increase in mean arterial blood pressure in* Pparα* ^−/−^ mice but did see a significantly greater increase in blood pressure to a high salt diet challenge than WT as well as distinct effects on renal tubules and kidney function [26]. Since blood pressure is a complex integration of multiple systems in the body, it is possible that different organs work to compensate to keep blood pressure regulated at basal conditions; however, blood pressure dysregulation due to impairment in* Pparα* ^−/−^ (or cardioprotection by PPAR*α* agonists) may be most prominent only during stressors such as that observed with Ang II or high salt.

PPAR*α* may play a particularly important role in the setting of diabetes. Low expression of PPAR*α* has been shown to be involved in microvascular complications of diabetic mice [15]. In diabetic rat aortas, treatment with PPAR*α* activators was able to restore the endothelium-dependent relaxations [28], and activation of PPAR*α* in type 2 diabetic patients displayed improved flow-mediated endothelium-dependent vasodilation as well as attenuation of oxidative stress [29–32]. Interestingly, Tordjman et al. demonstrated that* Pparα* deficiency in apoE-null mice fed a high fat diet resulted in higher atherogenic lipoproteins but lower fasting levels of glucose, reduced insulin resistance, and fewer atherosclerotic lesions. There was no statistical difference in systolic blood pressure at baseline, but there was a reduced blood pressure increase in response to the high fat diet in the* Pparα*/*apoE* deficient animals [33]. The authors suggested that reduced fatty acid oxidation in blood vessel walls of* Pparα* deficient mice could ultimately promote reduced superoxide production and thus decrease atherosclerosis and blood pressure. Nevertheless, we found that superoxide levels were increased in* Pparα* ^−/−^ vessels, which may indicate that other mechanisms are at play under these specific conditions. These results highlight the complicated relationship between fat metabolism and transport, glucose metabolism, endothelial function, kidney and liver responses, and blood pressure regulation, which requires additional research to tease out the role of PPAR*α* in the regulation of blood flow and blood pressure under different stressors.

## 5. Conclusions

Our findings highlight the significant role PPAR*α* may play in cardiovascular disease via suppression of ROS and maintenance of endothelial function. PPAR*α* could be an important therapeutic target for maintenance of the health of the endothelium, especially with age, increased inflammation, or diseases that alter endothelial function such as diabetes.

## References

[1] Staels B., Koenig W., Habib A. (1998). Activation of human aortic smooth-muscle cells is inhibited by PPARalpha but not by PPARgamma activators. *Nature*.

[2] Hiukka A., Maranghi M., Matikainen N., Taskinen M.-R. (2010). PPAR*α*: an emerging therapeutic target in diabetic microvascular damage. *Nature Reviews Endocrinology*.

[3] Taverne Y. J. H. J., Bogers A. J. J. C., Duncker D. J., Merkus D. (2013). Reactive oxygen species and the cardiovascular system. *Oxidative Medicine and Cellular Longevity*.

[4] Tabernero A., Schoonjans K., Jesel L., Carpusca I., Auwerx J., Andriantsitohaina R. (2002). Activation of the peroxisome proliferator-activated receptor alpha protects against myocardial ischaemic injury and improves endothelial vasodilatation. *BMC Pharmacology*.

[5] Cervantes-Pérez L. G., Ibarra-Lara M. D. L. L., Escalante B. (2012). Endothelial nitric oxide synthase impairment is restored by clofibrate treatment in an animal model of hypertension. *European Journal of Pharmacology*.

[6] Silswal N., Parelkar N. K., Wacker M. J., Brotto M., Andresen J. (2011). Phosphatidylinositol 3,5-bisphosphate increases intracellular free Ca^2+^ in arterial smooth muscle cells and elicits vasocontraction. *The American Journal of Physiology—Heart and Circulatory Physiology*.

[7] Andresen J. J., Shafi N. I., Durante W., Bryan R. M. (2006). Effects of carbon monoxide and heme oxygenase inhibitors in cerebral vessels of rats and mice. *The American Journal of Physiology—Heart and Circulatory Physiology*.

[8] Bryan R. M., You J., Phillips S. C. (2006). Evidence for two-pore domain potassium channels in rat cerebral arteries. *American Journal of Physiology—Heart and Circulatory Physiology*.

[9] Parelkar N. K., Silswal N., Jansen K., Vaughn J., Bryan R. M., Andresen J. (2010). 2,2,2-Trichloroethanol activates a nonclassical potassium channel in cerebrovascular smooth muscle and dilates the middle cerebral artery. *Journal of Pharmacology and Experimental Therapeutics*.

[10] Silswal N., Touchberry C. D., Daniel D. R. (2014). FGF23 directly impairs endothelium-dependent vasorelaxation by increasing superoxide levels and reducing nitric oxide bioavailability. *The American Journal of Physiology— Endocrinology and Metabolism*.

[11] Silswal N., Parelkar N. K., Wacker M. J., Badr M., Andresen J. (2012). PPAR*α*-independent arterial smooth muscle relaxant effects of PPAR*α* agonists. *PPAR Research*.

[12] Beyer A. M., de Lange W. J., Halabi C. M. (2008). Endothelium-specific interference with peroxisome proliferator activated receptor gamma causes cerebral vascular dysfunction in response to a high-fat diet. *Circulation Research*.

[13] Halabi C. M., Beyer A. M., de Lange W. J. (2008). Interference with PPAR*γ* function in smooth muscle causes vascular dysfunction and hypertension. *Cell Metabolism*.

[14] d’Uscio L. V., He T., Santhanam A. V. R., Tai L.-J., Evans R. M., Katusic Z. S. (2014). Mechanisms of vascular dysfunction in mice with endothelium-specific deletion of the PPAR-delta gene. *The American Journal of Physiology—Heart and Circulatory Physiology*.

[15] Hu Y., Chen Y., Ding L. (2013). Pathogenic role of diabetes-induced PPAR-*α* down-regulation in microvascular dysfunction. *Proceedings of the National Academy of Sciences of the United States of America*.

[16] Ismail-Beigi F., Craven T., Banerji M. A. (2010). Effect of intensive treatment of hyperglycaemia on microvascular outcomes in type 2 diabetes: an analysis of the ACCORD randomised trial. *The Lancet*.

[17] Sharma A., Sellers S., Stefanovic N. (2015). Direct endothelial nitric oxide synthase activation provides atheroprotection in diabetes-accelerated atherosclerosis. *Diabetes*.

[18] Madigan M., Zuckerbraun B. (2013). Therapeutic potential of the nitrite-generated NO pathway in vascular dysfunction. *Frontiers in Immunology*.

[19] Goya K., Sumitani S., Xu X. (2004). Peroxisome proliferator-activated receptor alpha agonists increase nitric oxide synthase expression in vascular endothelial cells. *Arteriosclerosis, Thrombosis, and Vascular Biology*.

[20] Fleenor B. S., Seals D. R., Zigler M. L., Sindler A. L. (2012). Superoxide-lowering therapy with TEMPOL reverses arterial dysfunction with aging in mice. *Aging Cell*.

[21] Ding L., Cheng R., Hu Y. (2014). Peroxisome proliferatore activated receptor a protects capillary pericytes in the retina. *The American Journal of Pathology*.

[22] Gunnett C. A., Chu Y., Heistad D. D., Loihl A., Faraci F. M. (1998). Vascular effects of LPS in mice deficient in expression of the gene for inducible nitric oxide synthase. *The American Journal of Physiology—Heart and Circulatory Physiology*.

[23] Briones A. M., Alonso M. J., Marin J., Salaices M. (1999). Role of iNOS in the vasodilator responses induced by L-arginine in the middle cerebral artery from normotensive and hypertensive rats. *British Journal of Pharmacology*.

[24] Gunnett C. A., Lund D. D., McDowell A. K., Faraci F. M., Heistad D. D. (2005). Mechanisms of inducible nitric oxide synthase-mediated vascular dysfunction. *Arteriosclerosis, Thrombosis, and Vascular Biology*.

[25] Smeets P. J. H., Teunissen B. E. J., Willemsen P. H. M. (2008). Cardiac hypertrophy is enhanced in PPAR alpha-/- mice in response to chronic pressure overload. *Cardiovascular Research*.

[26] Obih P., Oyekan A. (2008). Regulation of blood pressure, natriuresis and renal thiazide/amiloride sensitivity in PPAR*α* null mice. *Blood Pressure*.

[27] Wilson J. L., Duan R., El-Marakby A., Alhashim A., Lee D. L. (2012). Peroxisome proliferator activated receptor- agonist slows the progression of hypertension, attenuates plasma interleukin-6 levels and renal inflammatory markers in angiotensin II infused mice. *PPAR Research*.

[28] Olukman M., Sezer E. D., Ülker S., Sözmen E. Y., Çınar G. M. (2010). Fenofibrate treatment enhances antioxidant status and attenuates endothelial dysfunction in streptozotocin-induced diabetic rats. *Experimental Diabetes Research*.

[29] Cheng A. Y. Y., Leiter L. A. (2008). PPAR-alpha: therapeutic role in diabetes-related cardiovascular disease. *Diabetes, Obesity and Metabolism*.

[30] Avogaro A., Miola M., Favaro A. (2001). Gemfibrozil improves insulin sensitivity and flow-mediated vasodilatation in type 2 diabetic patients. *European Journal of Clinical Investigation*.

[31] Evans M., Anderson R. A., Graham J. (2000). Ciprofibrate therapy improves endothelial function and reduces postprandial lipemia and oxidative stress in type 2 diabetes mellitus. *Circulation*.

[32] Playford D. A., Watts G. F., Best J. D., Burke V. (2002). Effect of fenofibrate on brachial artery flow-mediated dilatation in type 2 diabetes mellitus. *The American Journal of Cardiology*.

[33] Tordjman K., Bernal-Mizrachi C., Zemany L. (2001). PPAR*α* deficiency reduces insulin resistance and atherosclerosis in apoE-null mice. *The Journal of Clinical Investigation*.

